# Inter-Physician Variation in Follow-Up Colonoscopies after Screening Colonoscopy

**DOI:** 10.1371/journal.pone.0069312

**Published:** 2013-07-18

**Authors:** Christian Stock, Michael Hoffmeister, Berndt Birkner, Hermann Brenner

**Affiliations:** 1 Division of Clinical Epidemiology and Aging Research, German Cancer Research Center (DKFZ), Heidelberg, Germany; 2 Institute of Medical Biometry and Informatics, University of Heidelberg, Heidelberg, Germany; 3 Private Gastroenterology Practice, Munich, Germany; 4 Bavarian Association of Statutory Health Insurance Physicians , Munich, Germany; University of Munich, Germany

## Abstract

**Background and Aims:**

Surveillance is an integral part of the colorectal cancer (CRC) screening process. We aimed to investigate inter-physician variation in follow-up procedures after screening colonoscopy in an opportunistic CRC screening program.

**Methods:**

A historical cohort study in the German statutory health insurance system was conducted. 55,301 individuals who underwent screening colonoscopy in 2006 in Bavaria, Germany, and who were not diagnosed with CRC were included. Utilization of follow-up colonoscopies performed by the same physician (328 physicians overall) within 3 years was ascertained. Mixed effects logistic regression modelling was used to assess the effect of physicians and other potential predictors (screening result, age group, and sex) on re-utilization of colonoscopy. Physicians were grouped into quintiles according to individual effects estimated in a preliminary model. Predicted probabilities of follow-up colonoscopy by screening result and physician group were calculated.

**Results:**

The observed rate of follow-up colonoscopy was 6.2% (95% confidence interval: 5.9-6.4%), 18.6% (17.8-19.4%), and 37.0% (35.5-38.4%) after negative colonoscopy, low-risk adenoma and high-risk adenoma detection, respectively. All considered predictors were statistically significantly associated with follow-up colonoscopy. The predicted probabilities of follow-up colonoscopy ranged from 1.7% (1.4-2.0%) to 11.0% (10.2-11.7%), from 7.3% (6.2-8.5%) to 35.1% (32.6-37.7%), and from 17.9% (15.5-20.6%) to 56.9% (53.5-60.3%) in the 1^st^ quintile (lowest rates of follow-up) and 5^th^ quintile (highest rates of follow-up) of physicians after negative colonoscopy, low-risk adenoma and high-risk adenoma detection, respectively.

**Conclusions:**

This study suggests substantial inter-physician variation in follow-up habits after screening colonoscopy. Interventions, including organizational changes in CRC screening should be considered to reduce this variation.

## Introduction

Primary screening for colorectal cancer (CRC) by colonoscopy with polypectomy has been recommended and offered for the average-risk population in Germany and the United States (US) since 2002 [1]. In both countries, CRC screening is usually performed on an opportunistic basis, i.e. utilization depends on initiatives of individuals or individual encounters with health care providers [1,2].

After screening colonoscopy, additional examinations for surveillance of screen-detected colorectal adenomas are required in 20-30% of individuals [3–7]. A surveillance colonoscopy is considered appropriate after 3 years in case of high-risk adenomas and after 5 years in case of low-risk adenomas [6–8]. Exceptions requiring earlier additional examinations are incompletely removed adenomas at screening and referral for polypectomy (of large adenomas) to hospitals or expert centers. Independently of screening and surveillance, additional colonoscopies might also be required if signs and symptoms of gastrointestinal diseases occur.

Surveillance is an integral part of the CRC screening process [9]. In order to ensure effective CRC screening, efficient resource use and minimal discomfort for patients, a high level of adherence to surveillance guidelines is desirable. Studies conducted in the US have often indicated overuse of surveillance colonoscopy in routine practice, particularly among low-risk individuals [10–14]. In a recent German study, 16% and 31% of screening colonoscopy participants had an additional procedure within 3 years and 5 years, respectively [15]. The reported rates of additional colonoscopy utilization are commonly averages among the entire screened population. However, the variation among physicians in follow-up habits and levels of adherence to surveillance guidelines has not been quantified in previous studies. This variation gives an indication of the quality of adenoma surveillance in practice and its knowledge would be of relevance for the design of possible intervention strategies to enhance guideline adherence.

The aim of this study was therefore to investigate inter-physician variation in follow-up procedures after screening colonoscopy in routine practice. 

## Methods

A historical cohort study of screening colonoscopy participants in Bavaria, Germany, was conducted. Participants were followed-up for additional colonoscopies performed by the same physician within 3 years after screening.

### Ethics statement

The ethics committee of the Medical Faculty at the University of Heidelberg has approved this study. Anonymized data routinely collected by health insurances were analyzed. Written informed consent of patients was infeasible and not required by the approving institutional review board.

### Data source

Data collected between 2006 and 2009 as part of a quality assurance program by the Bavarian Association of Statutory Health Insurance Physicians (“Qualitätsmaßnahme Koloskopie”) were used.

Bavaria is a federal German state situated in the south of Germany with a population of currently 12.5 million inhabitants (15% of the German population). The percentage of the population insured by the statutory health insurance (SHI) system in this time-period was 89%.

The database contains electronic colonoscopy documentation of screening and non-screening colonoscopies, including information on demographics, process quality, findings, complications, and diagnoses and treatments (as required by federal guidelines for screening colonoscopy). In case of non-screening colonoscopy, the indication for the procedure was recorded. In 2006, 86% of outpatient colonoscopies performed in the SHI system and 72% of all outpatient colonoscopies in Bavaria were included in this database [16]. Previous studies have used the database for cross-sectional analyses on quality of colonoscopy and risk of colorectal neoplasia [16–18]. For the longitudinal approach used in the present study, multiple colonoscopies per individual performed by the same physician were linked using physician identifiers (unique physician IDs in the SHI system) and physician-specific patient identifiers (unique patient IDs within the practice management system of each physician). Both types of identifiers had been recoded using a deterministic algorithm unknown to the analyst before being combined into one variable that allowed to link patient records within one practice management system.

To perform outpatient colonoscopies in the German SHI system, physicians need to fulfill professional and technical requirements. Only specialist physicians for internal medicine with subspecialization in gastroenterology or specialist physicians for surgery are allowed to perform colonoscopies. They are required to perform at least 200 total colonoscopies and 10 polypectomies per year. Quality control was performed using the image and video documentation of colonoscopies. A random sample of 20 colonoscopy documentations per physician and year was checked by an expert panel of gastroenterologists at the Bavarian Association of SHI physicians. It was required that 90% of the recorded diagnoses were valid based on the documentation.

### Eligibility criteria

The database was searched for all individuals undergoing screening colonoscopy in Bavaria in 2006 (N=59,871). Exclusion criteria were age <55 years (screening colonoscopy is not regularly offered earlier to asymptomatic people at average risk) (N=796), nonscreening colonoscopy prior to screening colonoscopy in 2006 (N=648), multiple screening colonoscopies per individual (N=2,206), carcinoma detected at screening (N=654). Individuals were also excluded if the corresponding physician documented <50 colonoscopies per year from 2006 to 2009 to avoid bias due to drop-out of physicians (N=4,266). After exclusion criteria were applied, the final cohort comprised 51,301 screening colonoscopy participants with 51,301 screening colonoscopies performed by 328 physicians.

### Variables

Utilization of additional colonoscopies performed within 3 years after screening colonoscopy by the same physician was ascertained.

Findings at screening colonoscopies were categorized as ‘negative’ (no adenomas, but including hyperplastic polyps), ‘low-risk adenoma’ (1-3 tubular adenomas, each <1cm, only low-grade intraepithelial neoplasia), and ‘high-risk adenoma’ (≥4 tubular adenomas, ≥1 adenoma ≥1 cm, adenoma with tubulo-villous or villous structure, high-grade intraepithelial neoplasia). Note that usually already ≥3 tubular adenomas are considered a high-risk adenoma situation. However, the data used for the present analysis did not allow to identify individuals with ≥3 adenomas because the categories used in the documentation were slightly different.

According to guidelines in force in 2006 in Germany, colonoscopic surveillance was recommended after 3 years in case of completely removed low-risk adenomas or high-risk adenomas [19]. In 2008, the national guideline was updated and the recommended surveillance interval for low-risk adenomas was extended to 5 years [20]. In case of negative screening colonoscopy, another screening colonoscopy is recommended after 10 years [7,19,20]. Regulations in the SHI system allow a second screening colonoscopy after 10 years if the first screening colonoscopy was performed below 65 years of age.

As factors potentially determining additional colonoscopy utilization, the physician who performed screening, age group (55-64, 65-74, 75+ years) and sex of screened individuals, and the screening result (negative colonoscopy, low-risk adenoma, high-risk adenoma) were considered.

If individuals presented with any of the following signs and symptoms of gastrointestinal diseases for additional colonoscopy, the indication was considered to be ‘any sign or symptom’: abdominal pain, anemia, change in bowel habits, diarrhea, incomplete defecation, incontinence, macroscopic bleeding, obstipation, painful defecation, perianal pain, positive fecal occult blood test, pruritus ani, weight loss, and other unspecified symptoms. Otherwise, the indication was considered to be ‘surveillance only’.

### Sensitivity analyses

In the main analysis, all follow-up colonoscopies were taken into account irrespective of their indication, i.e. surveillance or other diagnostic reasons (signs or symptoms). This was considered sensible for two reasons. First, surveillance or diagnostic reasons could be documented at the same time and often no “main” indication was available. Second, although, in terms of insurance claims no distinction is made in Germany between surveillance and other diagnostic colonoscopies and the specific reason is not required for billing purposes, partial misclassification of indications was deemed possible. For example, physicians aware of their non-adherence to guidelines when performing a colonoscopy primarily for surveillance but earlier than recommended could document other indications. Sensitivity analyses were conducted in which only additional colonoscopies done for surveillance only (no signs or symptoms) and at least partly for surveillance (possibly accompanied by signs or symptoms) were considered.

### Statistical methods

Percentages and corresponding 95%-confidence intervals (CI) of individuals with utilization of ≥1 follow-up colonoscopy were calculated stratified by screening result and age.

Mixed effects logistic regression modelling was applied to assess the variation among physicians in follow-up colonoscopies within 3 years after screening, adjusted for potential confounders age-group, sex and screening result.

In a preliminary logistic regression model of additional colonoscopy utilization, the explanatory variables age group, sex and screening result were included as fixed effects and individual physicians were included as random effects. Based on the random effect estimates obtained in this model, physicians were grouped into quintiles, with the 1^st^ quintile representing the 20% of physicians with the lowest follow-up rates and the 5^th^ quintile representing the 20% of physicians with the highest follow-up rates. The categorization into quintiles was chosen based on considerations of statistical power and interpretability.

The logistic regression model was then re-estimated with physician-group and the interaction between physician group and screening result as additional fixed effects. From this updated model, population-averaged probabilities of follow-up colonoscopy by physician group (quintiles) and screening result were obtained. An interaction term (between physician group and screening result) was required to compute predicted probabilities and corresponding variances. Similar approaches which aim to compare the quality across treatment centers have recently been grouped under the keyword “provider profiling”. [21]

All statistical tests were two-sided using a significance level of 0.05. The analyses were conducted using SAS 9.2 (SAS Institute Inc., Cary, North Carolina, USA). PROC GLIMMIX was used for mixed effects logistic regression models. The LSMEANS statement was applied to calculate predicted probabilities of follow-up colonoscopy. 

## Results

### Study population

The study population consisted of 51,301 individuals (56% females) who underwent screening colonoscopy in 2006. The mean age was 64.5 years (standard deviation: 6.8 years). In 75%, 17% and 8% of individuals, no adenomas, low-risk adenomas and high-risk adenomas were detected, respectively. Further characteristics are shown in Table 1.

**Table 1:** Study population.

**Characteristic**	**N**	**%**
Overall	51,301	100.0
Females	28,682	55.9
Age group		
55-64 years	26,785	52.2
65-74 years	19,923	38.8
75+ years	4,593	9.0
Complete screening colonoscopy	49,974	97.4
Result of screening colonoscopy		
Negative	38,340	74.7
Low-risk adenoma	8,679	16.9
High-risk adenoma	4,282	8.3
Polypectomy^a^	15,267	29.8

^a^ Polypectomy of adenomatous polyps (low-/ high-risk adenomas) or hyperplastic polyps (negative colonoscopy).

### Utilization of follow-up colonoscopy

The observed overall utilization of follow-up colonoscopy within 3 years was 6.2% (95%-CI: 5.9-6.4%), 18.6% (95%-CI: 17.8-19.4%), and 37.0% (95%-CI: 35.5-38.4%) after negative colonoscopy, low-risk adenoma, and high-risk adenoma, respectively. Utilization of follow-up colonoscopy was broadly similar across age groups as shown in Table 2.

**Table 2:** Utilization of follow-up colonoscopy within 3 years after screening colonoscopy.

**Result of screening colonoscopy**	**Age group**	**N**	**Additional colonoscopy within 3 years% (95% CI)**
Negative	55-64 years	20,683	5.6 (5.2, 5.9)
	65-74 years	14,362	6.9 (6.5, 7.3)
	75+ years	3,295	6.7 (5.8, 7.5)
	Total	38,340	6.2 (5.9, 6.4)
Low-risk adenoma	55-64 years	4,215	19.7 (18.5, 20.9)
	65-74 years	3,665	18.1 (16.9, 19.4)
	75+ years	799	14.9 (12.4, 17.4)
	Total	8,679	18.6 (17.8, 19.4)
High-risk adenoma	55-64 years	1,887	37.6 (35.4, 39.8)
	65-74 years	1,896	37.7 (35.5, 39.8)
	75+ years	499	32.1 (28.0, 36.2)
	Total	4,282	37.0 (35.5, 38.4)
Total	55-64 years	26,785	10.0 (9.7, 10.4)
	65-74 years	19,923	11.9 (11.5, 12.4)
	75+ years	4,593	10.9 (10.0, 11.8)
	Total	51,301	10.8 (10.6, 11.1)

Abbreviation: CI, confidence interval

**Table 3:** Predictors of follow-up colonoscopy within 3 years after screening colonoscopy.

**Characteristic**	**Mixed effects logistic regression model 1 (preliminary model)**		**Mixed effects logistic regression model 2 (final model)**	
*Fixed effects*	*OR (95% CI)*	*P value*	*OR (95% CI)*	*P value*
Physician group^a^				
Quintile 1	-	-	1.00 Ref.	<0.0001
Quintile 2	-	-	1.79 (1.57, 2.04)	
Quintile 3	-	-	2.54 (2.24, 2.88)	
Quintile 4	-	-	3.57 (3.15, 4.05)	
Quintile 5	-	-	6.73 (5.96, 7.60)	
Screening result				
Negative colonoscopy	1.00 Ref.	<0.0001	1.00 Ref.	<0.0001
Low-risk adenoma	4.00 (3.72, 4.31)		4.10 (3.78, 4.44)	
High-risk adenoma	10.26 (9.46, 11.13)		10.84 (9.95, 11.81)	
Age group				
55-64 years	1.00 Ref.	0.0002	1.00 Ref.	0.0005
65-74 years	1.04 (0.98, 1.11)		1.04 (0.98, 1.11)	
75+ years	0.82 (0.73, 0.92)		0.81 (0.73, 0.91)	
Sex				
Female	1.00 Ref.	<0.0001	1.00 Ref.	<0.0001
Male	1.14 (1.08, 1.22)		1.15 (1.08, 1.22)	
*Random effect*	*Variance onlogit scale (SE)*	*P value*	*Variance onlogit scale (SE)*	*P value*
Physician	0.52 (0.05)	<0.0001	0.07 (0.003)	0.001

a Physicians were categorized into quintiles according to random effect estimates obtained by the preliminary model.

Abbreviations: CI, confidence interval; OR, odds ratio, Ref., reference; SE, standard error.

### Predictors of follow-up colonoscopy

In the preliminary model, all fixed-effects variables (screening result, age group and sex) and the random physician effects were statistically significantly associated with follow-up colonoscopy as shown in Table 3. Especially screen-detected high-risk adenomas were associated with strongly increased odds of additional colonoscopy. Male sex was associated with a modest increase and high age (75+ years) with a modest decrease in the odds of additional colonoscopy.

**Figure 1:**
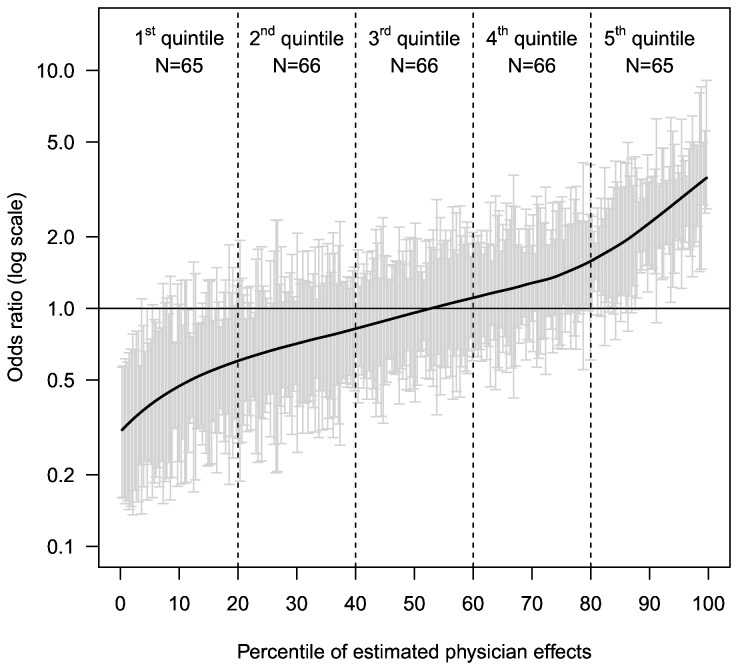
Estimated random physician effects on utilization of follow-up colonoscopy within 3 years after screening colonoscopy. The straight black line is a local polynomial regression line through the individual effect estimates. The error bars reflect 95%-confidence intervals.

Individual physician effects on re-utilization ranged from ORs of 0.30 (95%-CI: 0.16-0.57) to 4.89 (95%-CI: 2.63-9.09) and are displayed in Figure 1. One physician who contributed 179 screening colonoscopies (not shown in Figure 1) was excluded in further analyses due to a data quality issue. The resulting 5 physician groups accounted for 10,034, 9,709, 10,067, 9,917, and 11,395 screening colonoscopies in quintiles 1 to 5, respectively.

Inclusion of the physician group effect in the final model led to minor changes in the estimated ORs compared with the preliminary model, while the variance in the individual physician effects was expectedly smaller, as shown in Table 3. OR estimates for additional colonoscopy within 3 years increased up to 6.73 (95% CI: 5.96-7.60) in the 5^th^ quintile (highest rates of follow-up) compared to the 1^st^ quintile of physicians (lowest rates of follow-up). The physician group effects were very similar when only colonoscopies done for surveillance were considered (Table S1).

### Differences between physicians

**Figure 2:**
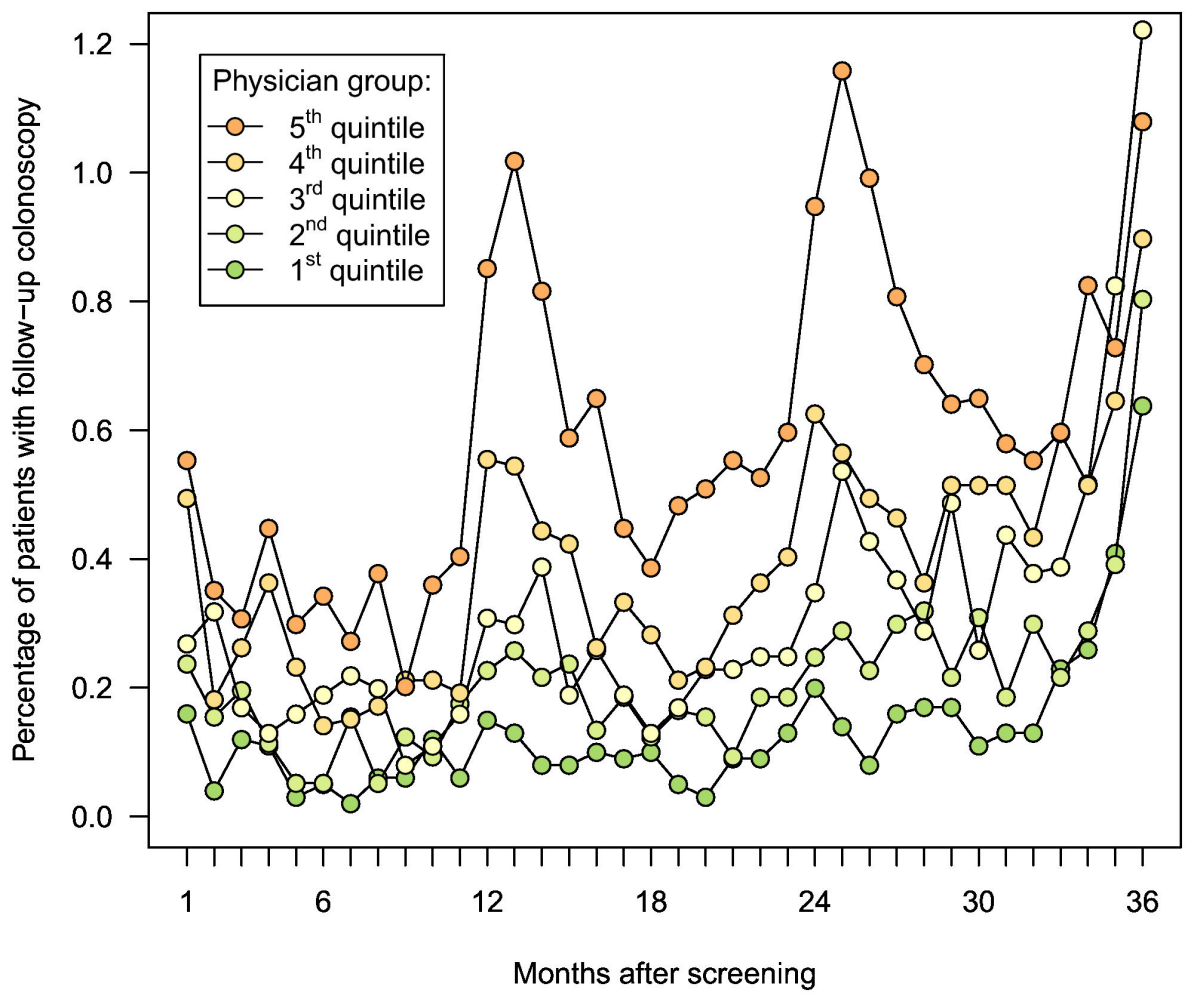
Observed monthly utilization of follow-up colonoscopy within 3 years after screening colonoscopy according to physician group.

The utilization of follow-up colonoscopy within 3 years after screening according to physician group is shown in Figure 2. Especially in the group with the highest rates of follow-up (quintile 5), peaks in colonoscopy utilization were observed around 12, 24 and 36 months after screening. For all groups the utilization increased shortly before the end of the observation period.

The fraction of follow-up colonoscopies performed for symptoms, as opposed to surveillance, was 38%, 38%, 37%, 42% and 46% in quintiles 1 to 5, respectively. The median number of colonoscopies in 2006 (i.e. screening and non-screening colonoscopies) among physicians in quintiles 1 to 5 was 443, 550, 516, 542 and 525, respectively, while the mean fraction of screening examinations per physician ranged between 25% and 27% in the 5 groups.

**Figure 3:**
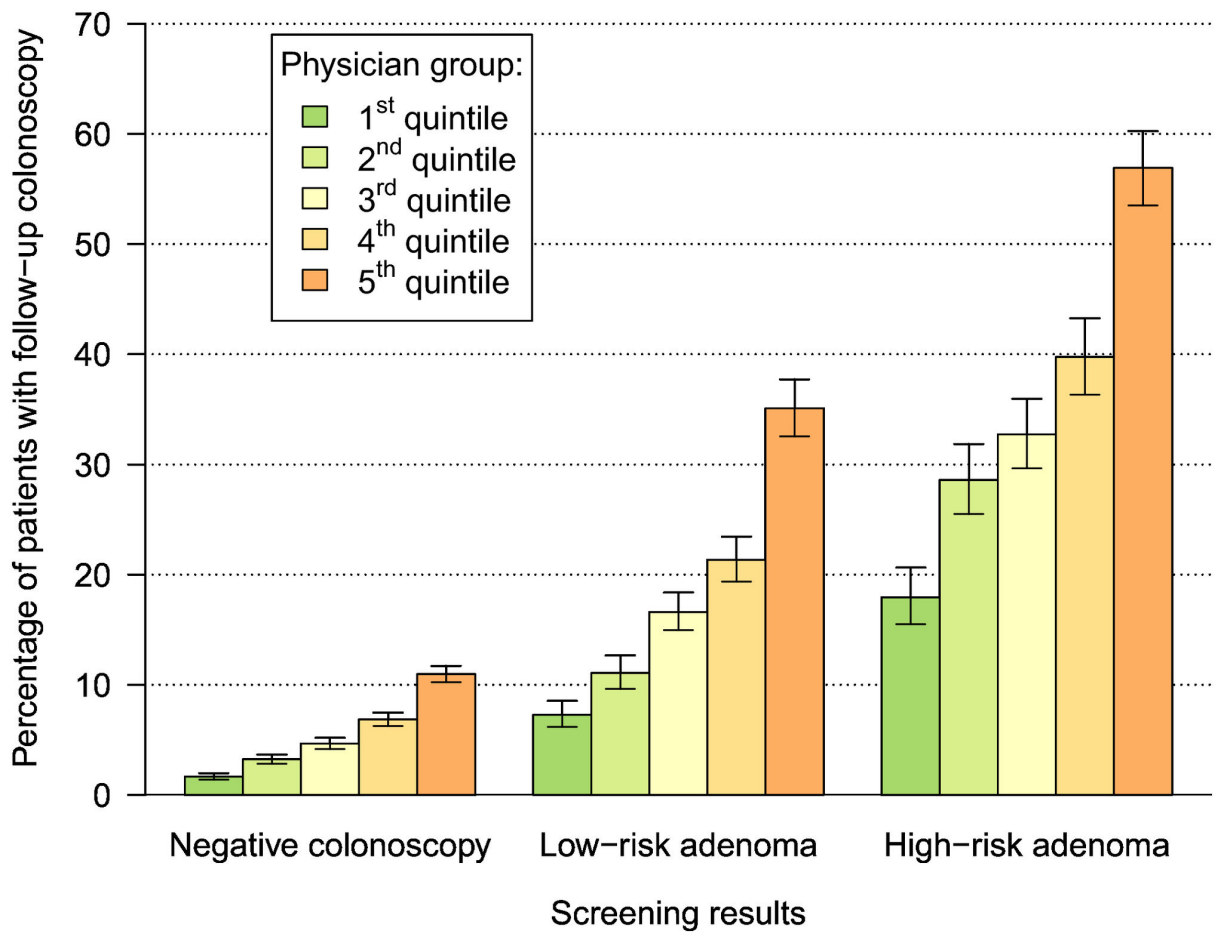
Predicted probability of follow-up colonoscopy within 3 years after screening colonoscopy according to screening result and physician group. The error bars reflect 95%-confidence intervals.

The predicted probabilities of follow-up colonoscopy (hereafter expressed as percentages) ranged from 1.7% (1.4-2.0%) to 11.0% (10.2-11.7%), from 7.3% (6.2-8.5%) to 35.1% (32.6-37.7%), and from 17.9% (15.5-20.6%) to 56.9% (53.5-60.3%) in the 1^st^ quintile (lowest rates of follow-up) and 5^th^ quintile (highest rates of follow-up) of physicians after negative colonoscopy, low-risk adenoma, and high-risk adenoma, respectively. All predicted probabilities are depicted in Figure 3. When only surveillance indications were considered in sensitivity analyses, the range of predicted probabilities was considerably lower after negative screening colonoscopy, but it remained very substantial after screen-detected low- or high-risk adenomas (Table S2).

## Discussion

In this study, inter-physician variation in follow-up examinations after screening colonoscopy was investigated. The analysis was conducted in the German healthcare system and is based on a large colonoscopy documentation database reflecting routine practice in the time period from 2006 to 2009.

A substantial fraction of screening colonoscopy participants underwent additional colonoscopy with the same physician in the subsequent 3 years for either surveillance or symptoms. Besides expected variation in frequency of additional colonoscopy according to screening results, large variation between physicians was observed. Predicted probabilities of re-utilization according to screening result and physician group, defined by quintiles of re-colonoscopy frequencies, ranged from 1.7% to 11.0%, 7.3% to 35.1%, and from 17.9% to 56.9% in the different groups after negative colonoscopy, low-risk adenoma, and high-risk adenoma, respectively. This variation remained very substantial in sensitivity analyses when only documented surveillance indications were considered. Approximately three out of five colonoscopies were conducted for surveillance as opposed to symptoms.

Independent of the screening result, generally no surveillance colonoscopy is recommended in the first 3 years after screening, the time period considered in the present study. While many of the early repeat colonoscopies shortly after screening may have been performed because of problems at screening, e.g. insufficient bowel preparation or incomplete polypectomy, most colonoscopies shortly before the end of the observation period are likely to have been performed for surveillance in accordance with guidelines.

Overuse of surveillance colonoscopy was indicated by peaks in utilization rates after 12 months and 24 months following screening which most likely reflect frequent recommendations of surveillance colonoscopy after one or two years, respectively. These peaks were very prominent especially in the 5^th^ quintile, i.e. among the physicians with the highest follow-up proportions.

Inter-physician variation in follow-up colonoscopies is a phenomenon that may be typically observed in opportunistic CRC screening programs, where utilization of services depends on the initiative of individuals and on individual encounters with health care providers [2]. Naturally, recommendations for surveillance and performance of additional colonoscopy due to symptoms will differ to some extent between physicians due to different levels of guideline awareness and adherence, as well as different levels of experience. By contrast, in organized programs, invitations to screening and usually also to surveillance are issued from centralized registers. It appears very plausible that usage of a central database of screening outcomes and central invitations to surveillance colonoscopy is likely to result in less inter-physician variation and better overall adherence to adenoma surveillance guidelines.

From a public health perspective, substantial variation in use of surveillance colonoscopy in a CRC screening program is not desirable. Whether the magnitude of the variation in follow-up colonoscopy is acceptable and whether interventions within the system of opportunistic screening (e.g. to improve guideline awareness and adherence, or patient–physician communication) or even a change of the screening system might be justified, is to be decided by gastroenterologists and health policy-makers. As a consequence of widespread use of colonoscopy for CRC screening in an aging population, surveillance colonoscopy will pose an increasing financial burden to healthcare-payers in future. Strategies that ensure efficient use of resources are therefore required. A recent German study suggested that surveillance colonoscopies after previous colorectal adenomas constituted approximately 11% of the total number of colonoscopies performed in 2006 [22]. In the United States, already a decade ago, surveillance colonoscopy after removal of adenomas was the single most common indication for colonoscopy in patients older than 50 years, accounting for 15% of procedures in women and 22% of procedures in men [23]. The impact of too early surveillance colonoscopies and variation among physicians on the cost-effectiveness of CRC screening is uncertain. This study indicates that imperfect adherence to surveillance guidelines should be considered in economic evaluations of CRC screening practice.

Internationally, there has so far been little research into utilization, predictors and outcomes of follow-up examinations after screening colonoscopy. Most of the available studies were conducted in the United States where CRC screening is usually also performed on an opportunistic basis. They generally indicate overutilization of surveillance colonoscopy in low-risk situations, but partly also underutilization among higher-risk individuals [12,24]. In a Medicare-based study, early repeat colonoscopy within 1 year was more frequent if the index examination had been performed by a family physician, general surgeon or internist compared with a gastroenterologist, and it was less frequent if it was performed by an endoscopist in the lower quartiles of colonoscopy volume [13]. An economic decision-modelling study concluded that aggressive surveillance can be expensive or even harmful [25]. A survey among US-gastroenterologists found that surveillance colonoscopy was recommended much more frequently than indicated according to clinical guidelines, especially for hyperplastic polyps and small adenomas [10]. Although physicians partly lacked knowledge about guideline recommendations for post-polypectomy surveillance, even those who were aware of the recommendations often ignored them and performed surveillance colonoscopy sooner than recommended [11].

To our knowledge, this study is the first large-scale investigation addressing inter-physician variation in performance of follow-up colonoscopies after screening colonoscopy. Strengths of this study from Germany include the very large database and high coverage of the population of a large federal state with a population of more than 10 million people. The present findings reflect routine practice and may in principle be generalizable to the German SHI system (covering ~90% of the German population) where different incentives to perform and to utilize health services exist for physicians and patients.

The methodological approach used here employs random effects from a mixed effects logistic regression model for grouping of physicians and prediction of quality-related group-specific outcomes. A provider profile is obtained which is adjusted for the case-mix of patients in terms of age, sex and screening result. This method generally appears useful to investigate variation in healthcare practice and outcomes, especially with databases that include a large number physicians (or alternatively: treatment centers, hospitals, etc.) and also a large number of procedures per provider. It might also be used to facilitate detection of quality issues and benchmarking among providers. For example, in the field of colorectal cancer screening, a further application could be an assessment of the variation in adenoma detection rates among physicians.

Several limitations need to be considered in the interpretation of the present results. First, the study is an analysis based on colonoscopy documentation data not primarily collected for research purposes. Second, only follow-up colonoscopies conducted by the same physician who had done the screening colonoscopy could be identified and were included. The overall frequency of additional colonoscopies should be higher than reported here. However, the interest in the present analysis was in the variation in follow-up habits of the physicians, not in an estimate of the overall utilization of further colonoscopies. Third, only a small number of physician- and patient-level factors were available. Additional physician-level factors, such as experience, specialization, age and gender, would have helped to better explain inter-physician variation in follow-up procedures after screening colonoscopy. Patient level factors, such as comorbidities, family history of cancer and behavioral risk factors would have been useful to further adjust for the case-mix of physicians beyond the available factors age, sex and screening result. Finally, as all follow-up colonoscopies were considered in the main analysis (not only those for surveillance) due to risk of misclassification bias, the results are based on the assumption that the likelihood of presenting with signs or symptoms of gastrointestinal diseases is equally distributed in the age-, sex- and adenoma-subgroups of patients across physicians. Unobserved patient-level covariates that are clustered for physicians and have an impact on colonoscopy utilization could partly also explain variability that was attributed to physicians here. 

## Conclusion

In summary, this large-scale investigation into the utilization of follow-up colonoscopies suggests substantial inter-physician variation in follow-up habits after screening colonoscopy in Germany, a country with widespread opportunistic screening colonoscopy. Although the introduction of screening colonoscopy was accompanied by efforts of quality assurance which might have contributed to the strong protection from CRC and low rates of interval cancers recently disclosed in a large case-control study from Germany [26–28], there seems to be room for improvement in quality assurance regarding adherence to surveillance guidelines. Interventions or organizational changes in the screening system should be considered to improve overall adherence to surveillance guidelines.

## Supporting Information

Table S1 **Predictors of surveillance colonoscopy within 3 years after screening colonoscopy.**
(PDF)

Table S2 **Predicted probability of follow-up colonoscopy within 3 years after screening.**
(PDF)
