# Supplementary material for: Inter-Physician Variation in Follow-Up Colonoscopies after Screening Colonoscopy
Source: PLoS One. 2013 Jul 18;8(7):e69312. doi: 10.1371/journal.pone.0069312 (PMC3715496; doi:10.1371/journal.pone.0069312)
Supplement: Table S1 — Predictors of surveillance colonoscopy within 3 years after screening colonoscopy. (PDF) [file pone.0069312.s001.pdf]

Table S1. Predictors of surveillance colonoscopy within 3 years after screening colonoscopy.

**Characteristic**	**Mixed effects logistic regression models**			
	**Surveillance only (no signs or symptoms)**		**Surveillance (partly accompanied by signs or symptoms)**	
Fixed effects	*OR (95% CI)*	*P value*	*OR (95% CI)*	*P value*
Physician group[a)]				
Quintile 1	1.00 Ref.	<0.0001	1.00 Ref.	<0.0001
Quintile 2	1.96 (1.54, 2.48)		1.81 (1.47, 2.24)	
Quintile 3	2.94 (2.35, 3.69)		2.83 (2.32, 3.46)	
Quintile 4	3.83 (3.06, 4.80)		3.70 (3.04, 4.51)	
Quintile 5	6.51 (5.21, 8.13)		6.52 (5.36, 7.92)	
Screening result				
Negative colonoscopy	1.00 Ref.	<0.0001	1.00 Ref.	<0.0001
Low-risk adenoma	9.08 (8.04, 10.26)		9.53 (8.52, 10.65)	
High-risk adenoma	25.68 (22.72, 29.04)		25.83 (23.04, 29.00)	
Age group				
55-64 years	1.00 Ref.	<0.0001	1.00 Ref.	<0.0001
65-74 years	0.93 (0.85, 1.01)		0.94 (0.87, 1.01)	
75+ years	0.69 (0.59, 0.80)		0.69 (0.60, 0.79)	
Sex				
Female	1.00 Ref.	<0.0001	1.00 Ref.	<0.0001
Male	1.25 (1.16, 1.36)		1.21 (1.13, 1.32)	
Random effect	*Variance on logit scale (SE)*	*P value*	*Variance on logit scale (SE)*	*P value*
Physician	0.13 (0.02)	<0.0001	0.09 (0.02)	<0.0001

The interaction effect between screening result and physician group included in the final model (surveillance only: P=0.07; surveillance: P=0.009) was omitted from this table for simplicity. It led to minor deviations in the individual effects of the variables.

[a] Physicians were categorized into quintiles according to random effect estimates obtained by the preliminary model (see Table 3).

Abbreviations: CI, confidence interval; OR, odds ratio, Ref., reference; SE, standard error.
