# Supplementary material for: Inter-Physician Variation in Follow-Up Colonoscopies after Screening Colonoscopy
Source: PLoS One. 2013 Jul 18;8(7):e69312. doi: 10.1371/journal.pone.0069312 (PMC3715496; doi:10.1371/journal.pone.0069312)
Supplement: Table S2 — Predicted probability of follow-up colonoscopy within 3 years after screening. (PDF) [file pone.0069312.s002.pdf]

Table S2. Predicted probability of follow-up colonoscopy within 3 years after screening.

**Screening result**	**Physician group**[a)]				
	**Quintile 1 (lowest)**	**Quintile 2**	**Quintile 3**	**Quintile 4**	**Quintile 5 (highest)**
	**% (95% CI)**	**% (95% CI)**	**% (95% CI)**	**% (95% CI)**	**% (95% CI)**
Probability of any follow-up colonoscopy [b)]					
Negative colonoscopy	1.7 (1.4, 2.0)	3.2 (2.8, 3.7)	4.7 (4.2, 5.2)	6.8 (6.3, 7.5)	11.0 (10.2, 11.7)
Low-risk adenoma	7.3 (6.2, 8.5)	11.1 (9.6, 12.7)	16.6 (15.0, 18.4)	21.3 (19.4, 23.4)	35.1 (32.6, 37.7)
High-risk adenoma	17.9 (15.5, 20.6)	28.6 (25.5, 31.9)	32.7 (29.7, 36.0)	39.8 (36.3, 43.3)	56.9 (53.5, 60.3)
Probability of follow-up colonoscopy for surveillance (possibly accompanied by signs or symptoms)					
Negative colonoscopy	0.5 (0.4, 0.7)	1.0 (0.8, 1.3)	1.9 (1.6, 2.3)	2.5 (2.1, 2.9)	3.8 (3.3, 4.4)
Low-risk adenoma	5.7 (4.7, 6.9)	8.7 (7.3, 10.4)	13.8 (12.0, 15.8)	16.9 (14.8, 19.2)	28.7 (25.7, 31.8)
High-risk adenoma	15.3 (12.8, 18.1)	24.6 (21.4, 28.2)	28.2 (24.9, 31.8)	34.1 (30.4, 38.1)	48.1 (44.0, 52.2)
Probability of follow-up colonoscopy for surveillance only (no signs or symptoms)					
Negative colonoscopy	0.4 (0.3, 0.6)	0.9 (0.7, 1.1)	1.6 (1.3, 2.0)	2.1 (1.7, 2.5)	3.2 (2.7, 3.7)
Low-risk adenoma	4.5 (3.6, 5.6)	7.3 (6.0, 8.8)	11.6 (9.9, 13.5)	13.9 (11.9, 16.1)	24.0 (21.1, 27.2)
High-risk adenoma	12.9 (10.6, 15.6)	22.4 (19.1, 26)	24.5 (21.3, 28.1)	30.6 (26.8, 34.6)	42.0 (37.8, 46.4)

[a] Physicians were categorized into quintiles according to random effects estimated by mixed effecs logistic regression model 1 (Table 3).

[b] Displayed in Figure 3.

Abbreviations: CI, confidence interval.
